# Online Self-Determination Toolkit for Youth With Disabilities: Protocol for a Mixed Methods Evaluation Study

**DOI:** 10.2196/20463

**Published:** 2021-01-11

**Authors:** Sally Lindsay, Polina Kosareva, Mahadeo Sukhai, Nicole Thomson, Jennifer Stinson

**Affiliations:** 1 Bloorview Research Institute Holland Bloorview Kids Rehabilitation Hospital and University of Toronto Toronto, ON Canada; 2 Department of Occupational Science & Occupational Therapy University of Toronto Toronto, ON Canada; 3 Canadian National Institute for the Blind Toronto, ON Canada; 4 Center for Addiction and Mental Health Toronto, ON Canada; 5 Lawrence Bloomberg Faculty of Nursing and Hospital for Sick Children Toronto, ON Canada

**Keywords:** disability, involvement, occupational therapy, rehabilitation, youth

## Abstract

**Background:**

Youth with disabilities encounter many challenges during their transition to adulthood including finding employment. Jobs are often inaccessible, and youth often face a lack of support, discriminatory attitudes, and sometimes low self-confidence. Therefore, it is critical to help youth enhance their self-determination skills to advocate for their needs in the workplace.

**Objective:**

The aim of this paper is to describe how an online toolkit aimed to improve self-determination in advocating for needs, including disability disclosure and accommodation requests to employers, was co-created with youth with disabilities.

**Methods:**

We will use a mixed method design in which qualitative data (ie, focus groups and mentored discussion forum) are collected to understand the contextual factors during the intervention that could affect outcomes or explain results through the pre-post questionnaires. Fifty youths with disabilities aged 15 to 24 years will be recruited.

**Results:**

Data collection is in progress. Planned analyses include focus groups and pre-post surveys to determine the impact of the intervention on self-determination. A qualitative content analysis of the focus groups and all open-ended survey questions will be conducted to understand the impact of the toolkit.

**Conclusions:**

Our online toolkit includes evidence-informed content that was co-created with youth who have a disability. It has potential for educational and vocational programming for youth with disabilities.

**International Registered Report Identifier (IRRID):**

PRR1-10.2196/20463

## Introduction

### Background

Although there is substantial evidence that people with disabilities are a strong asset to our workforce, their employment rates are persistently lower than people without disabilities [1]. This trend is especially true for youth with disabilities, who have significantly lower employment rates compared with youth without disabilities. For example, the employment rate for youth in Canada aged 20 to 24 years with a severe disability is 35% and youth with a mild or moderate disability is 57% compared with 87% of youth without a disability [2]. For youth aged 15 to 19 years with disabilities, their employment rates are 40% compared with 51% of youth without disabilities [2]. Some research shows that significantly more youth with disabilities leave high school and remain unemployed compared with youth without disabilities [3,4]. Therefore, it is critical to provide them with support and tools to enhance their employment outcomes.

### Employment and Youth With Disabilities

There are approximately 540,000 Canadians aged 15 to 24 years who have disabilities [5]. Such youth represent a unique population facing a challenging transition to adulthood and are at an increased risk for poor health outcomes and psychological distress [6-8]. Many youths, especially those with disabilities, often find the transition to adulthood and securing work to be difficult [9-12]. Although there are often many stereotypes about people with disabilities, many of them, including youth, are willing and able to work, yet they remain one of the most marginalized groups in the labor force [10,11]. Research consistently shows that having a disability can be an obstacle to finding work, where people encounter difficulties at both the societal (ie, stigma and discrimination, inaccessible jobs) and individual (ie, low self-confidence) level [4,9,13-16]. As a result of such barriers, youth with disabilities commonly have higher unemployment rates compared with youth without disabilities [17]. Focusing on youth and young adults with disabilities is particularly worthwhile because they often have difficulties with developmental tasks, social development, and role functioning [6,18]. Additionally, this period of emerging adulthood (ie, 18 to 25 years) is characterized by identity exploration, instability, and development of executive functioning, all factors that are critical for achieving independence and securing employment [6]. This developmental period is an optimal time to enhance positive behaviors while developing work-based identities [6,8,10].

### Workplace Disability Disclosure and Accommodation

In many countries, workplace accommodations (eg, modified environment, flexible hours, adaptive technology) are supported by human rights and accessibility legislation that places a duty on employers to provide reasonable accommodations to employees with disabilities [19,20]. Disclosure of a disability or health condition is a prerequisite to receiving workplace accommodations [21-23], which have potential to improve work participation and well-being [24,25]. However, research shows that many people with disabilities are reluctant to tell an employer about their condition because they are concerned about potential discrimination or job loss [9,11,25,26]. For example, some 55% of Canadians with disabilities believe that hiding their disability increases their chances of getting hired and promoted [17]. Such high rates of nondisclosure are concerning because working without accommodations can hinder health, quality of life, and work productivity [27-29]. Youth may be particularly reluctant to disclose their disability to an employer given their inexperience and/or engagement in precarious work (ie, part-time, casual, or contract work) [4]. Although many people with disabilities could benefit from having workplace accommodations, only a small proportion of employees are disclosing their needs [30-33]. This trend is concerning, especially for youth with disabilities who often have unique developmental needs; workplace policies that are typically implemented for adults may be inappropriate for youth [30,33]. Therefore, providing them with additional support on how they could advocate for their needs may be worthwhile.

### Self-Determination and Youth With Disabilities

Youth with disabilities often have lower levels of self-confidence and self-determination (ie, attitudes and abilities required to act as the primary agent in one’s life to make choices free from external influence [34]) compared with youth without disabilities [35], which are important factors that can affect employment outcomes. This trend often results from having fewer opportunities to develop self-advocacy and independence skills than youth without disabilities. For individuals to develop self-determination they need to establish coping behaviors when facing adverse situations (eg, rejection from employers, unemployment) through having a consistent effort and exposure to social learning experiences. Some studies have shown that having high self-efficacy can lead to positive outcomes compared with having lower self-efficacy [36]. For example, Bandura [37] explains that self-efficacy can be improved through mastery of experience, social modeling, verbal persuasion, and improving physical and emotional states. Other research has shown that students with disabilities who have high self-determination have favorable employment outcomes, access to job benefits, and financial independence 1 to 3 years after postsecondary graduation [38]. Indeed, self-determination is often a predictor of students’ transition outcomes 2 years after graduation [16]. Given the importance of self-determination in enhancing employment outcomes, the aim of our study is to develop an online toolkit to optimize self-determination of youth with disabilities, specifically helping them to advocate for their needs and consider the pros and cons of disclosing their condition and requesting workplace accommodations.

### Online Toolkit to Enhance Self-Determination

One potential way to address vocational possibilities for youth with disabilities is through an online toolkit to enhance self-determination. Toolkits offer a way to package multiple knowledge translation (KT) strategies that educate and facilitate behavior change [39] and outcomes [40]. KT toolkits provide a simple, more flexible method for promoting and using best practices [40]. To be effective, toolkits should provide high-quality evidence to guide their use or implementation and have a planned approach and active engagement [39,40]. Combining online resources with interactive KT strategies may increase the likelihood of successful outcomes in evidence-based practice knowledge, skills, and behavior [41,42]. Our toolkit is an online interactive PDF that includes a PowToon video (PowToon Ltd), Articulate 360 (Articulate Global Inc) e-learning, and simulations.

### Theoretical Framework: Social Cognitive Career Theory

Learning and behavior change are greatly influenced by how well a message is heard, understood, and trusted and how much support individuals receive in translating new knowledge into changing practices [43,44]. We draw on social cognitive career theory to inform our understanding of youths’ development of self-determination after using our toolkit to enhance disability disclosure. This theory is an expansion of Bandura’s [37] psychological theory of social cognition that focuses on cognitive and motivational processes. In the context of vocational psychology, this theory was expanded to include career development [45]. This theory focuses on how people make work decisions, develop interests, and cope with work-related barriers and involves three main constructs: self-efficacy (ie, a person’s belief about their capability to organize and execute courses of action that are required to attain a type of performance), outcome expectations (ie, personal beliefs about the consequences of performing particular behaviors), and personal goals (ie, determination to participate in an activity or affect a future outcome) [37,46]. Each of these constructs is foundational toward achieving independence and work-related goals. Having goals allows individuals to exercise personal agency while contributing to enhanced self-efficacy in work-related roles [47].

Social cognitive career theory considers the ways in which individuals acquire and maintain behavior while also incorporating the social environment in which individuals perform behaviors [37]. The theory also considers a person’s past experiences that influence whether behavioral action will occur. Such past experiences can influence reinforcements and expectations that affect whether a person will engage in specific behaviors and the reasons for their engagement [37].

Within the context of our intervention, we hypothesize that the online toolkit will help increase self-determination. Specifically, when an individual has strong self-determination skills (ie, capability of executing behaviors) and expects a positive or successful outcome they will be more inclined to form goals for sustaining or increasing their participation in an activity [47]. Using a theory-framed approach [48], specifically drawing on the social cognitive career theory, will help to provide context for us to consider how our online toolkit might influence self-determination for youth with disabilities.

## Methods

### Objectives

The primary objective of this project is to describe how an online toolkit was co-created with youth with disabilities to improve their self-determination and describe the evaluation plan.

### Design

We will use a mixed method design in which qualitative data (ie, focus groups with a pre-post survey and mentored discussion forum) are collected to understand the contextual factors during the intervention that could affect outcomes or explain results (through pre-post questionnaires) [49,50]. The rationale, design, and content of our intervention are based on several systematic reviews that focused on the benefits of hiring people with disabilities [1], workplace disclosure and accommodation requests for youth with disabilities [51,52], the role of gender in finding and maintaining employment among youth with disabilities [53], vocational interventions for youth with disabilities [54], mentorship programs to facilitate transition to employment for youth with disabilities [55], and a review of electronic mentoring programs and interventions for youth with disabilities [56]. Needs assessments of youth with disabilities, employers (eg, disability awareness/confidence), and clinicians regarding disclosure and accommodations were also conducted in an earlier phase of this study [57-60]. There are currently no online toolkits that address work-related self-determination for this population.

Institutional ethical approval and informed written consent will be obtained from all participants prior to starting. We will follow the best practices in developing, implementing, and evaluating user-centered content as a KT strategy [39-42,61,62] including the consolidated framework for implementation research [63].

### Procedures for Development of Youth Toolkit Intervention

The purpose of the intervention is to improve the self-determination of youth with disabilities through an online toolkit about disability disclosure and workplace accommodations. The intervention, which was co-created with two youths who have disabilities (one with a visible physical disability and one with an acquired brain injury) along with a knowledge user advisory group and evidence-informed content from our team. The youths were recruited as paid project staff (ie, youth facilitators) and received appropriate training in research and project-specific training. We wrote the toolkit in youth-friendly, lay language. Several sections and tools within the toolkit were written and co-designed by youths. We then had an additional three youths with disabilities review our toolkit for usefulness and comprehensiveness. The youths also provided suggestions for layout and graphic design. After incorporating their suggestions, we had it reviewed by our hospital’s health literacy committee. Finally, our team reviewed the content, layout, and graphics at all stages with youths.

### Interactive and Immersive Learning Tools

We codeveloped interactive and immersive learning tools with youth with disabilities that included information about what a disability disclosure is and why it is important, things to consider before disclosing, how and when to ask for workplace accommodations, learning to self-advocate, knowing your rights (including a PowToon video co-designed with youth), and a words of advice section (including youth and employer case studies). We also included two simulations.

#### PowToon Animated Video

Our previous systematic reviews and needs assessments revealed that youth with disabilities wanted more information about workplace rights [51,57]. Therefore, we developed an interactive tool that specifically addressed this (Multimedia Appendix 1). We had a youth with a disability (ie, a paid member of our team) design a PowToon, a user friendly web-based animated video tool. The youth first researched relevant content with input from the research team. They then developed a script, which was audio recorded and used voiceover with the video. Next, graphics were added to the sound and slides were created with visuals from PowToon. Finally, the timing and appearance of all the elements were adjusted accordingly. The final version of the video will be embedded within the appropriate section of the toolkit.

#### Articulate Storyline

Another tool within the online toolkit involved Articulate 360, which is a multimedia platform and e-learning authoring tool. A youth with a disability wrote the story based on their lived experience with looking for a job and returning to work after an acquired brain injury (Multimedia Appendix 2). They sought feedback from the research team and scripted the story using the branching feature, which allows participants to follow different routes depending on their actions. We worked through the whole storyline as a team and assisted with the graphic design. Next, animated buttons were inserted so the previous and next slides could be triggered. Additionally, given that this is an interactive tool, layers for each slide were created so that when a participant clicks on a specific aspect of the slide, it would trigger a different response along with appropriate feedback. Finally, the text-to-speech tool was used to add audio.

#### Simulations

We integrated two simulations (ie, life-like environments and contrived social situations that mimic problems or conditions that arise in professional encounters) [64] into the toolkit. One focused on a youth with a disability in a job interview and the other one involved a recently hired youth with a disability who was asking their employer for workplace accommodations. By involving youth with disabilities in building the scenarios for these simulations, it helped to enhance their relevance and authenticity [64-66].

The simulation development sessions each lasted 2 hours and were facilitated by a researcher who is certified in SIM-One simulations (ie, briefing, debriefing, and facilitation). The first session focused on building the simulation scenario content (with three youths who have disabilities), and the second session was centered on piloting the scenario with live actors (ie, simulated participants) who trained for their character roles prior to participating in this session.

We asked the youth for feedback on the simulation content, its relevance for youth with disabilities, and any recommendations they had for further development. We then worked with a simulation educator and simulated participants to finalize a scenario script. We piloted the disability disclosure scenario with feedback from youth and the research team. We incorporated all feedback into the final version of the simulation, which was filmed and embedded within the toolkit.

#### Mentored Discussion Forum

The purpose of this intervention is to combine the youth toolkit with mentor-based learning, provided through a secure, online platform (myability.ca). This consists of case-based mentored discussions (ie, 50 participants total; 10 participants per group, plus mentor, across 5 groups). Each topic will cover the toolkits and simulations and other interactive materials. Trained mentors will lead discussions while encouraging interaction and support between participants. Mentors will be hired as project staff and will undergo appropriate training.

The mentored discussion forum consists of asynchronous discussions (ie, separate discussion for each main topic in the toolkit) aligned with the topics in the youth toolkit. Participants can view the discussions and the toolkit at their own pace, ask questions, and chat with the mentor and other participants. The discussion is open to the other participants in the group and available only to the participants in that particular group (ie, they cannot see participant discussions who are in another group). Participants can decide when they want to log in and contribute at a time that is convenient for them. Mentors will post their availability when they will be logged in so that participants have an opportunity to discuss things in real time.

Trained mentors will include near-peers (ie, young adults with a disability who have job experience) who have completed a youth mentor or equivalent training. Mentors will introduce the topics in the same order and will be trained to respond to participant comments in a similar manner—providing informational, appraisal, and emotional support. Prior to working with participants, mentors will practice their skills with fellow mentors and other research team members whose recent experiences will be similar to those of mentored participants (eg, training on active listening, perspective taking, confidentiality, maintaining boundaries, positive modeling, trust building through interactive training, and mentoring).

Mentors will lead discussions based on the youth toolkit over 2-week periods during the course of 6 months (each participant would only take part once). We would offer them at different times throughout the year to capture different youth (eg, summer break, March break). Mentors will have an opportunity to meet (virtually) before joining our project website.

### Sample and Recruitment

All participants will be recruited through invitation letters, referrals, or advertisements via our project partners. We will collaborate with our partners (Multimedia Appendix 3) and other relevant community agencies that help young people with disabilities find employment to identify eligible participants. Using such an approach to obtain a purposive sample has been effective in previous studies. We will recruit 50 youths (aiming for an equitable representation of genders) to take part in one of several focus groups (10 participants in each). Youth participants aged 15 to 24 years (based on the United Nations definition of youth) [67] will be included based on the following criteria: able to read/write in English; have a disability (ie, defined as impairments in body function or structure, activity limitations, and participation restrictions) and currently employed, enrolled in training, or seeking employment; and willingness to be audio recorded.

#### Focus Groups

After developing the content, we will host focus groups (2 hours each) with participants to gather feedback on the content, usability, and layout of the toolkits. During the focus groups, a researcher will give participants an opportunity to go through the toolkit and will answer any questions they may have. Participants will receive a link to the toolkit, which is hosted on the Holland Bloorview Kids Rehabilitation website, and/or a copy of the interactive PDF of the toolkit to review in advance of the focus group. We will go through each section with the youths, facilitated by a member of our research team, while asking them about the relevance and usefulness of each section. We will also ask about what they liked most and least, what they learned (if anything), and any other thoughts that they would like to discuss relevant to the toolkit and topic.

#### Pre-Post Surveys

After testing at our pilot sites and refining the toolkits based on feedback from the focus groups, we will work with our project partners to embed these educational tools as part of ongoing training (eg, youth employment and life skills programs), a strategy linked with higher likelihood of facilitating change [68]. We will train researchers and knowledge user champions to deliver the intervention offsite through our partners and networks. The toolkits and simulations will be available through our project website and linked to our project partners, where youth can self-refer to access the tool and health care providers can also direct youth to this resource. Participants using the toolkits and simulations will be asked to complete a brief online survey via Research Electronic Data Capture, and we will assess wider scale uptake [63].

### Feasibility and Sample Size

To test the primary hypothesis that our intervention will improve self-determination for youth with disabilities [69], a *t* test will be used. With an alpha of .05, power of 80%, and at least a medium effect size (ie, 0.50), a sample size of at least 50 is needed [70,71]. This sample size, which incorporates the possible attrition of participants, is suitable for this study design [72], and there are sufficient eligible participants to draw upon through our partners and collaborators.

### Data Collection

The data collection for this study is currently in progress.

### Measures

All standardized measures have good internal consistency, construct-related and criterion validity, and test-retest reliability and are widely used for people with disabilities. We will use Arc’s self-determination scale, a self-report measure assessing self-determination for disabled adolescents [69] (subscales on autonomy, self-realization, and psychological empowerment), and community participation (subscales related to workplace) [73].

#### Secondary Measures

Secondary measures for all participants will include demographic measures such as age, gender, type of disability, assistive devices, education level, and type of work. We will have open-ended questions asking what they liked most and least about the intervention. We will draw on the community impacts of research-oriented partnerships, subscales on personal knowledge and development [74], and subscales from the toolkit evaluation questionnaire by Malik et al [75]. To assess the simulations that are embedded within the toolkit, we will draw on some satisfaction questions from Kirkpatrick and Kirkpatrick [76] evaluating training programs. To measure the broader impacts of the interventions, we will use the consolidated framework for implementation research [63,77] and the reach, effectiveness, adoption, implementation, and maintenance (RE-AIM) framework [78]. Secondary qualitative data consists of open-ended questions in the pre- and postsurvey and transcripts of the focus groups and mentoring discussion forums.

## Results

Data collection for this study is in progress. The proposed analysis is outlined in further detail below.

### Quantitative Data Analyses

Quantitative data will be analyzed using SPSS Statistics (IBM Corp). Descriptive statistics provide an overview of the sample characteristics using means and standard deviations for continuous factors and frequencies and proportions for categorical factors. Chi-square and *t* test analyses will be conducted to test intervention effects (comparing preintervention survey primary outcomes, time 1) and posttest data (immediately postintervention, time 2). Separate analyses will be run for each outcome while exploring gender. To control for type I error rate, a Holm sequential correction will be applied. Effect sizes for *t* tests and Cohen *d* will be reported [70] with a level of .05 for statistical significance.

### Qualitative Data Analyses

All open-ended questions will be entered into NVivo (QSR International). Researchers will independently read all transcripts while noting key codes. Our objectives will guide the analysis, and data will be analyzed separately for focus groups and discussion forum data. An inductive, open-coding content analysis will be used [79] while specifically noting codes around disclosure, accommodations, mentoring, and disability awareness. Two researchers will read all transcripts to familiarize themselves with the data, generating initial codes, and revising and defining the codes and themes [79]. We will then meet to discuss our codes and revise them until consensus is reached among the research team on the final coding scheme (ie, separate ones for discussion forum and focus group data). We will apply all of the codes to the transcripts where they will be categorized into themes and subthemes. After the discussion forum and focus group transcripts are coded once in their entirety, they will be compared and contrasted using a qualitative comparative method [80] to see whether any differences within and between the groups appear. We will develop a thematic comparison table to help analyze what themes may be present in each group, and representative quotes will be extracted.

Strategies to ensure rigor and trustworthiness (ie, transferability, dependability, conformability) of the findings include prolonged engagement, peer debriefing, and descriptive participant accounts [81,82]. We will keep an audit trail of the decisions made during the analysis [79]. We will include excerpts from the transcripts that were reflective of the participant experiences to illustrate the themes [79]. We will also have peer debriefing discussions among the research team, which will include considering how our background training and experience may have influenced with the development of the themes while noting this in our audit trail [79].

## Discussion

### Principal Findings

This study will make several contributions to knowledge. First, most research on accommodations focuses on return to work among adults. Research focusing on workplace accommodations for youth with disabilities is lacking [30,52]. Second, more theoretically informed work is needed to support youth disclosing their condition through the development of evidence-informed interventions. Establishing innovative interventions that are scalable to a wide range of knowledge users could help to enhance disclosure discussions and inclusive environments, ultimately helping young workers to succeed in maintaining meaningful and productive employment.

### Conclusion

This intervention was co-created with youth with disabilities to help enhance their self-determination and self-advocacy skills in finding and maintaining employment. Helping youth with disabilities to develop such skills is important because they are an underrepresented group in the labor market. Our intervention may help youth to enhance their employment while helping them on their career pathway as they transition to adulthood. Our intervention can also serve as an accessible tool to supplement traditional vocational programming for youth with disabilities.

## Appendix

Multimedia Appendix 1 PowToon animation.

Multimedia Appendix 2 Articulate storyline.

Multimedia Appendix 3 Project partners.

## Abbreviations

KT: knowledge translation
RE-AIM: reach, effectiveness, adoption, implementation, maintenance
