# Supplementary material for: Online Self-Determination Toolkit for Youth With Disabilities: Protocol for a Mixed Methods Evaluation Study
Source: JMIR Res Protoc. 2021 Jan 11;10(1):e20463. doi: 10.2196/20463 (PMC7834931; doi:10.2196/20463)
Supplement: Multimedia Appendix 1 [file resprot_v10i1e20463_app1.pdf]

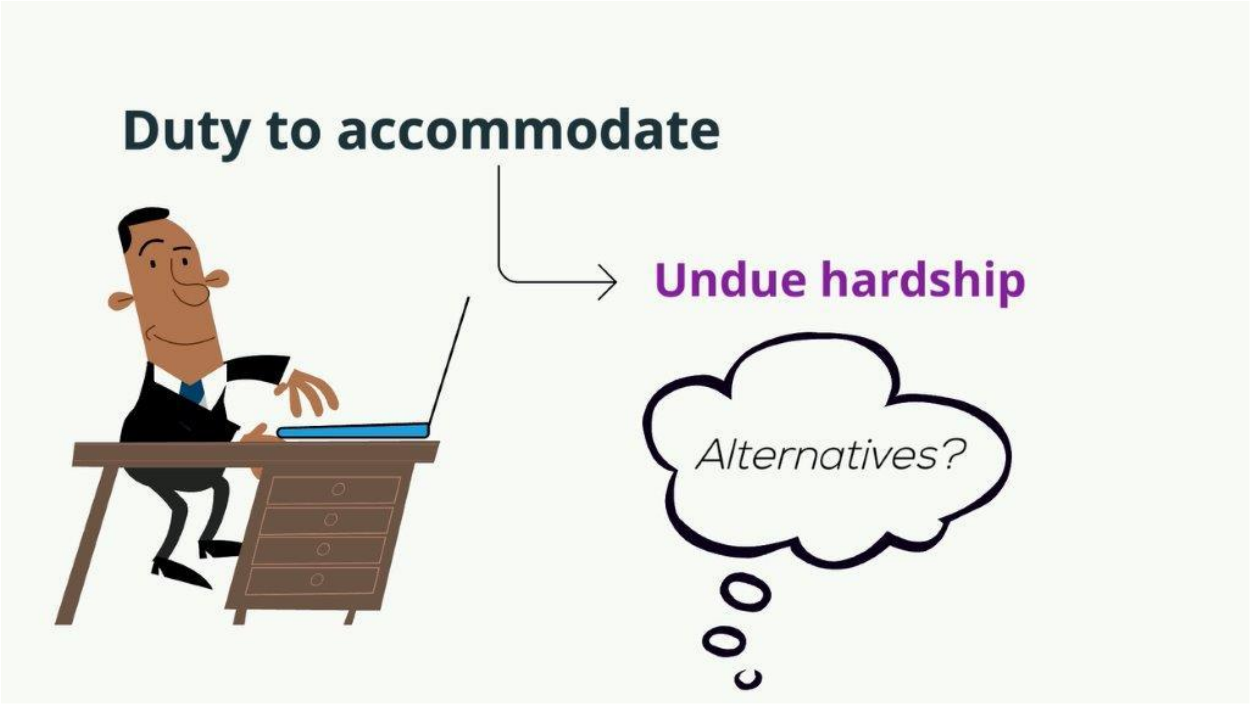
Duty to accommodate
Undue hardship
Alternatives?

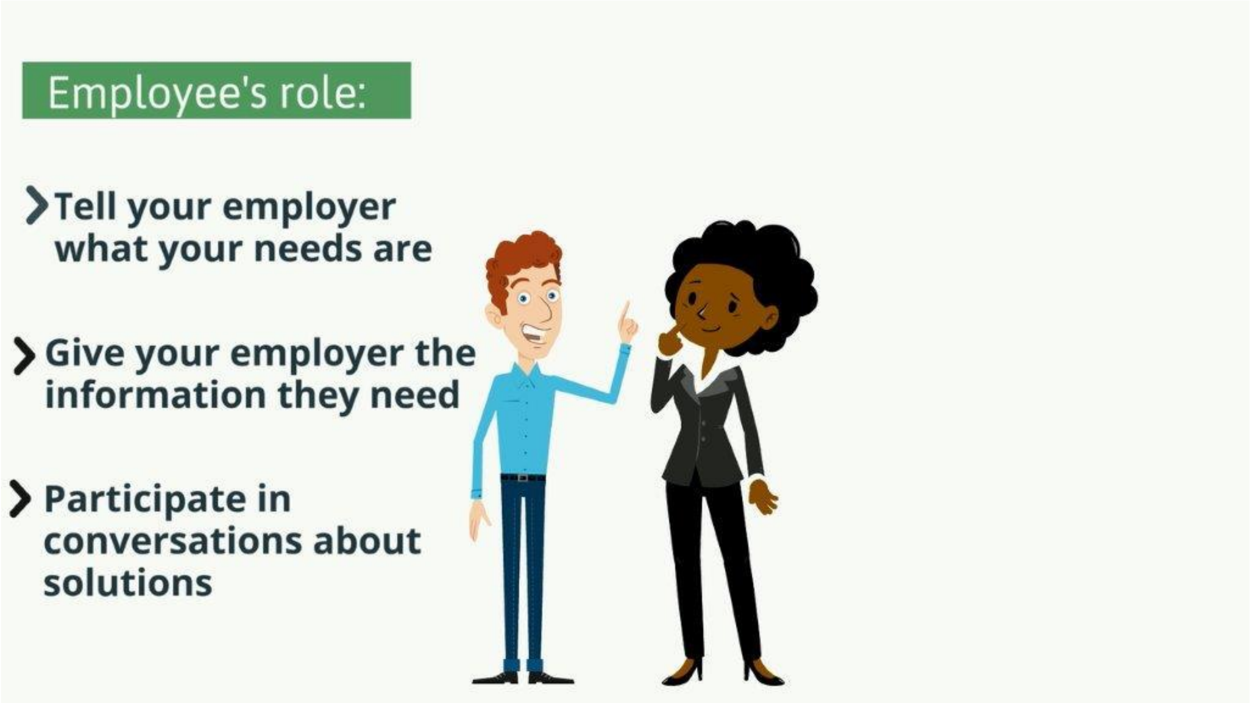
Employee's role:
Tell your employer what your needs are
Give your employer the information they need
Participate in conversations about solutions

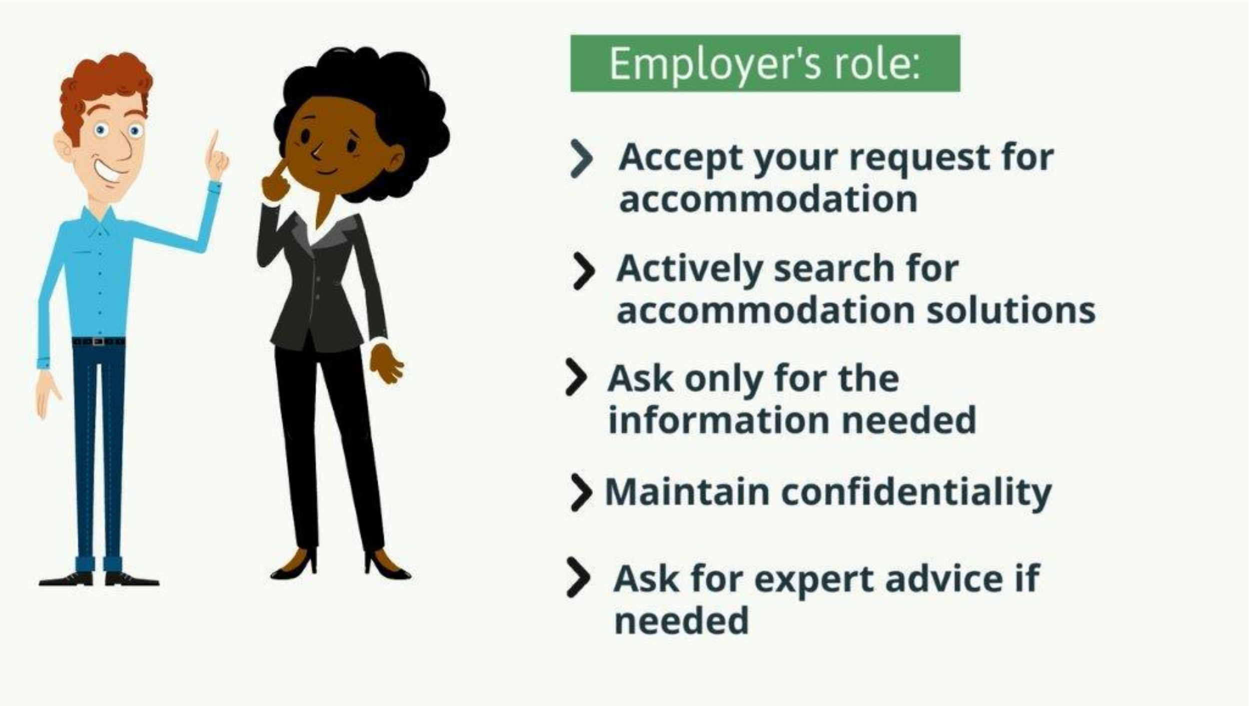
Employer's role:
Accept your request for accommodation
Actively search for accommodation solutions
Ask only for the information needed
Maintain confidentiality
Ask for expert advice if needed

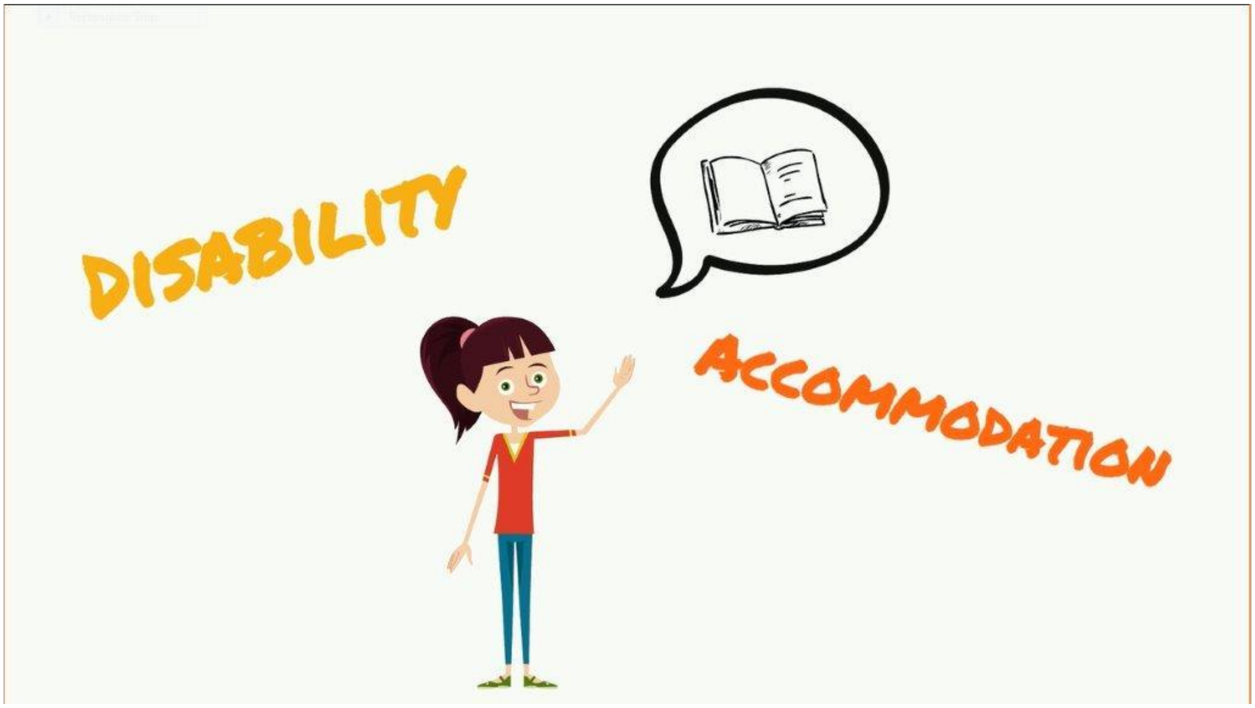
DISABILITY
ACCOMMODATION

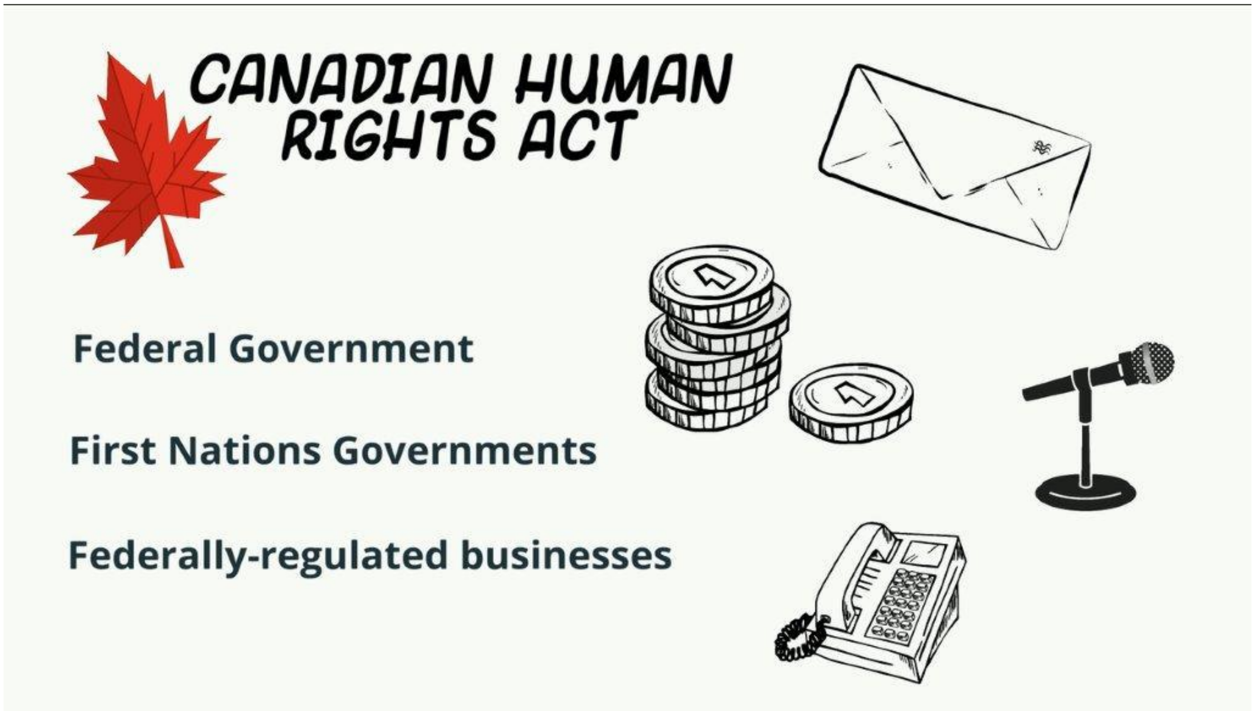
CANADIAN HUMAN RIGHTS ACT
Federal Government
First Nations Governments
Federally-regulated businesses
