# Supplementary material for: Online Self-Determination Toolkit for Youth With Disabilities: Protocol for a Mixed Methods Evaluation Study
Source: JMIR Res Protoc. 2021 Jan 11;10(1):e20463. doi: 10.2196/20463 (PMC7834931; doi:10.2196/20463)
Supplement: Multimedia Appendix 2 [file resprot_v10i1e20463_app2.pdf]

Articulate Storyline 360

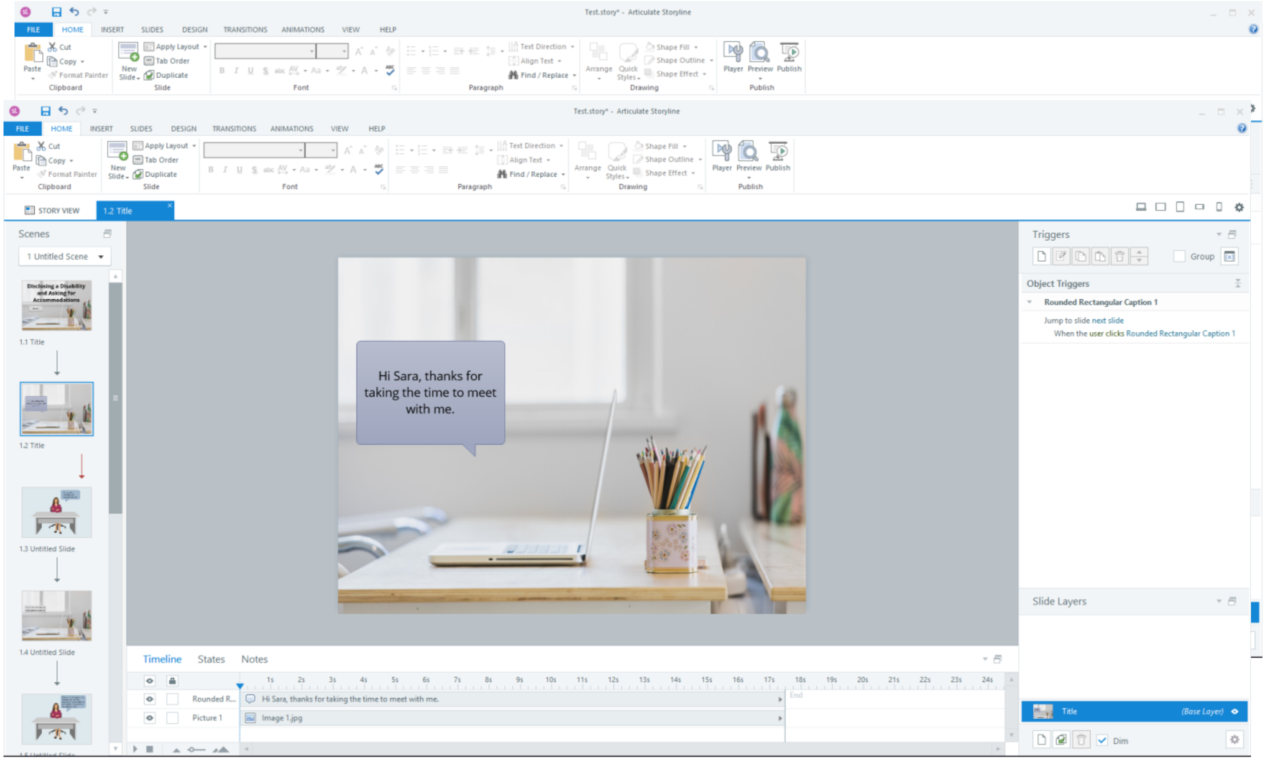

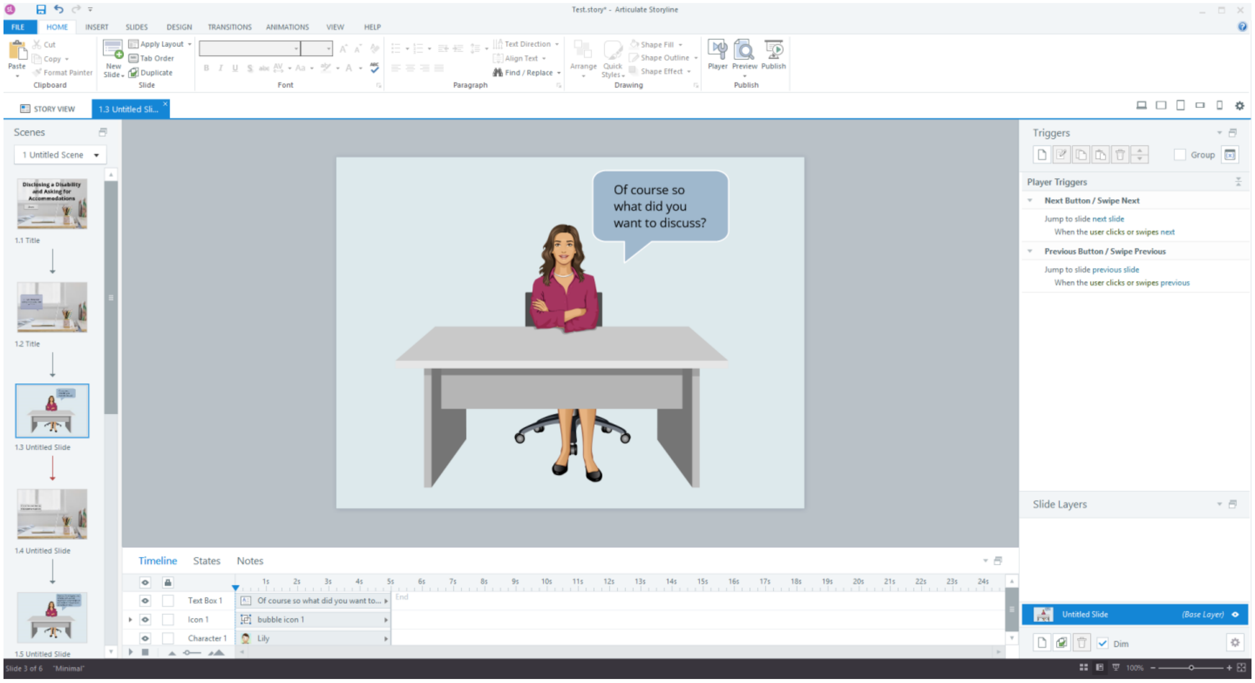
Test.story* - Articulate Storyline
FILE HOME INSERT SLIDES DESIGN TRANSITIONS ANIMATIONS VIEW HELP
STORY VIEW
1.3 Untitled Sli...
Scenes
1 Untitled Scene
1.1 Title
1.2 Title
1.3 Untitled Slide
1.4 Untitled Slide
1.5 Untitled Slide
Of course so what did you want to discuss?
Triggers
Player Triggers
Next Button / Swipe Next
Jump to slide next slide
When the user clicks or swipes next
Previous Button / Swipe Previous
Jump to slide previous slide
When the user clicks or swipes previous
Slide Layers
Untitled Slide
(Base Layer)
Dim
Timeline States Notes
Text Box 1
Of course so what did you want to...
Icon 1
bubble icon 1
Character 1
Lily
Slide 3 of 6 "Minimal"

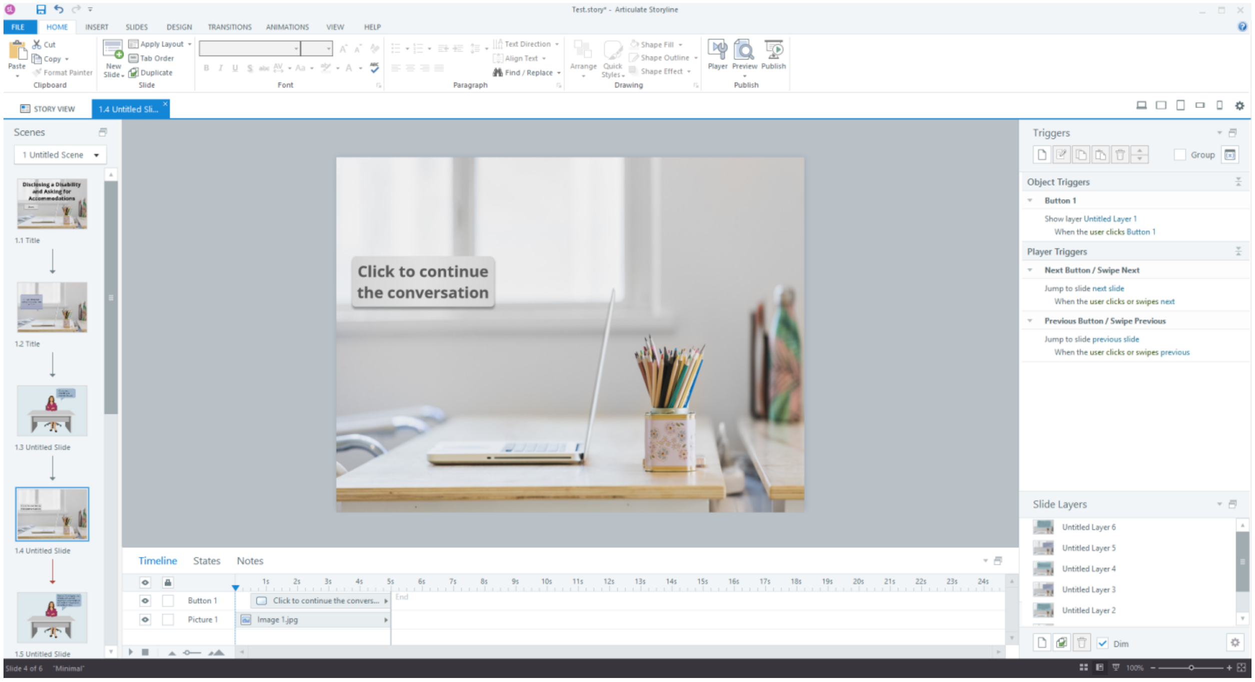
Test.story* - Articulate Storyline
FILE HOME INSERT SLIDES DESIGN TRANSITIONS ANIMATIONS VIEW HELP
STORY VIEW
1.4 Untitled Sli...
Scenes
1 Untitled Scene
1.1 Title
1.2 Title
1.3 Untitled Slide
1.4 Untitled Slide
1.5 Untitled Slide
Click to continue the conversation
Triggers
Object Triggers
Button 1
Show layer Untitled Layer 1
When the user clicks Button 1
Player Triggers
Next Button / Swipe Next
Jump to slide next slide
When the user clicks or swipes next
Previous Button / Swipe Previous
Jump to slide previous slide
When the user clicks or swipes previous
Slide Layers
Untitled Layer 6
Untitled Layer 5
Untitled Layer 4
Untitled Layer 3
Untitled Layer 2
Dim
Timeline States Notes
Button 1
Click to continue the convers...
Picture 1
Image 1.jpg
Slide 4 of 6 "Minimal"

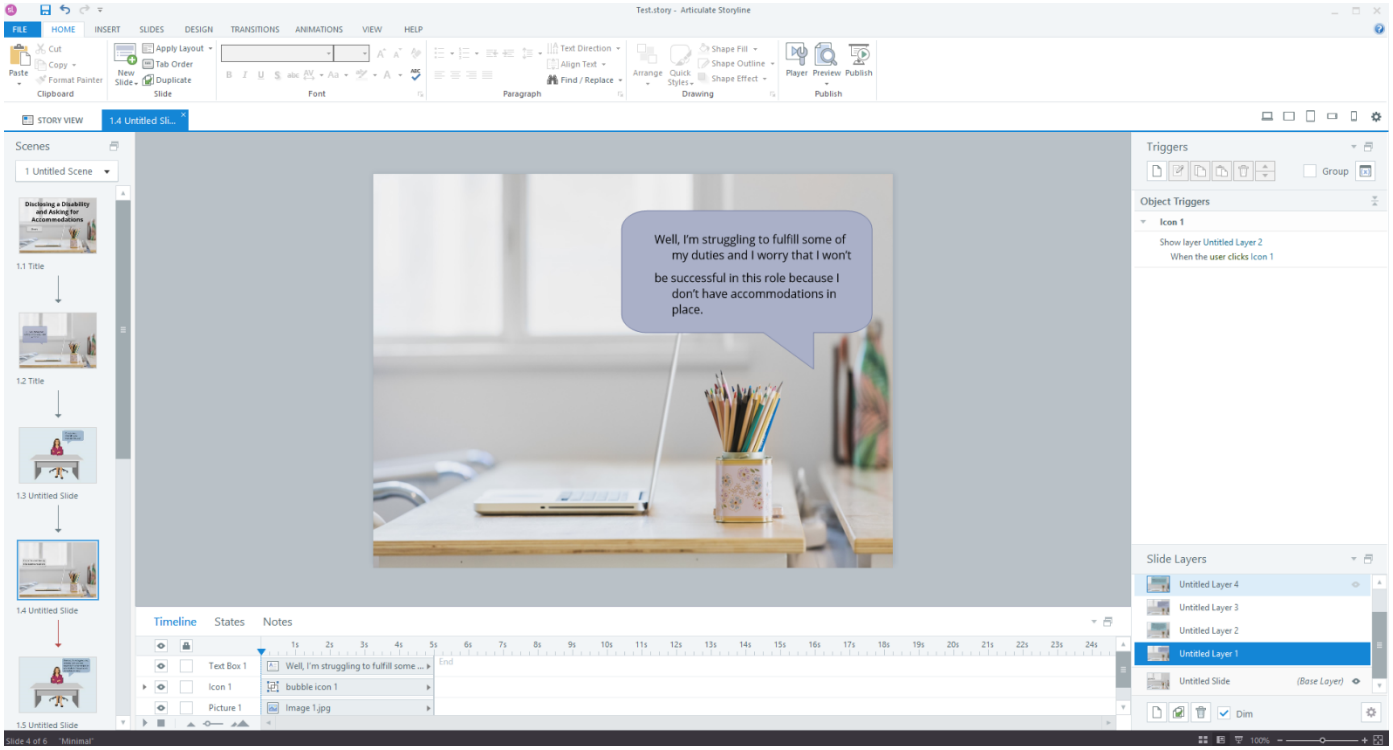
Test.story - Articulate Storyline
Well, I'm struggling to fulfill some of my duties and I worry that I won't
be successful in this role because I don't have accommodations in place.
Triggers
Object Triggers
Icon 1
Show layer Untitled Layer 2
When the user clicks Icon 1
Slide Layers
Untitled Layer 4
Untitled Layer 3
Untitled Layer 2
Untitled Layer 1
Untitled Slide
Timeline
States
Notes
Text Box 1
Icon 1
Picture 1
Scenes
1.1 Title
1.2 Title
1.3 Untitled Slide
1.4 Untitled Slide
1.5 Untitled Slide

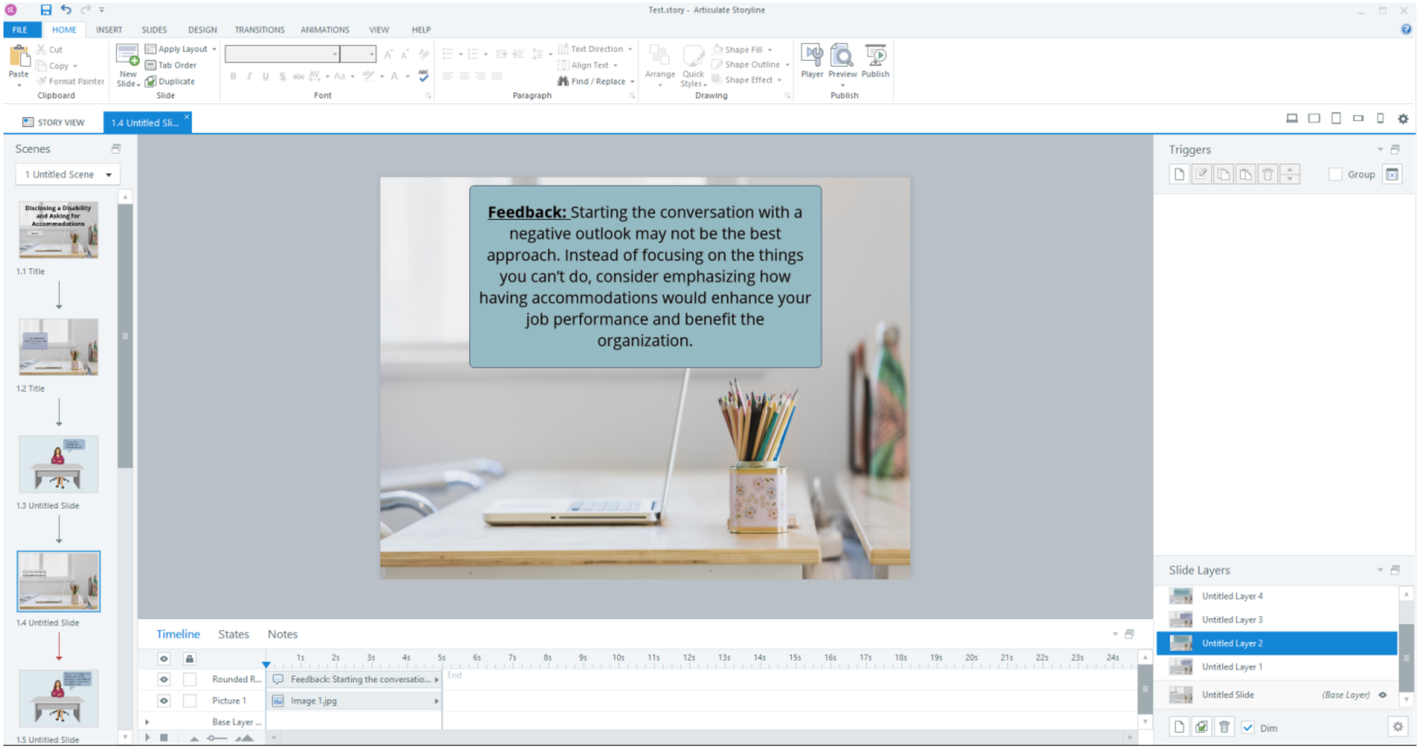
Test.story - Articulate Storyline
Feedback: Starting the conversation with a negative outlook may not be the best approach. Instead of focusing on the things you can't do, consider emphasizing how having accommodations would enhance your job performance and benefit the organization.
Triggers
Slide Layers
Untitled Layer 4
Untitled Layer 3
Untitled Layer 2
Untitled Layer 1
Untitled Slide
Timeline
States
Notes
Rounded R...
Picture 1
Base Layer ...
Scenes
1.1 Title
1.2 Title
1.3 Untitled Slide
1.4 Untitled Slide
1.5 Untitled Slide
