# Supplementary material for: Online Self-Determination Toolkit for Youth With Disabilities: Protocol for a Mixed Methods Evaluation Study
Source: JMIR Res Protoc. 2021 Jan 11;10(1):e20463. doi: 10.2196/20463 (PMC7834931; doi:10.2196/20463)
Supplement: Multimedia Appendix 3 [file resprot_v10i1e20463_app3.pdf]

Project Partners
Ability Online
Centre for Addiction and Mental Health (CAMH)
Canadian National Institute for the Blind Foundation
Canadian Council on Rehabilitation and Work
Dolphin Digital Technologies
Holland Bloorview Kids Rehabilitation Hospital
Hydrocephalus Canada
National Disability Mentoring Coalition (NDMC)
Pathways to Education (Unison Health & Community Services)
The Hospital for Sick Children (Sick Kids)
The University of Toronto
